# A Simplified Approach for the Molecular Classification of Glioblastomas

**DOI:** 10.1371/journal.pone.0045475

**Published:** 2012-09-18

**Authors:** Marie Le Mercier, Delfyne Hastir, Xavier Moles Lopez, Nancy De Nève, Calliope Maris, Anne-Laure Trepant, Sandrine Rorive, Christine Decaestecker, Isabelle Salmon

**Affiliations:** 1 Department of Pathology, Erasme University Hospital, Université Libre de Bruxelles (ULB), Brussels, Belgium; 2 Laboratory of Image Synthesis and Analysis (LISA), Brussels School of Engineering/École Polytechnique de Bruxelles, Université Libre de Bruxelles (ULB), Brussels, Belgium; 3 DIAPath - Center for Microscopy and Molecular Imaging (CMMI); Académie Universitaire Wallonie-Bruxelles, Gosselies, Belgium; NIH, United States of America

## Abstract

Glioblastoma (GBM) is the most common malignant primary brain tumors in adults and exhibit striking aggressiveness. Although GBM constitute a single histological entity, they exhibit considerable variability in biological behavior, resulting in significant differences in terms of prognosis and response to treatment. In an attempt to better understand the biology of GBM, many groups have performed high-scale profiling studies based on gene or protein expression. These studies have revealed the existence of several GBM subtypes. Although there remains to be a clear consensus, two to four major subtypes have been identified. Interestingly, these different subtypes are associated with both differential prognoses and responses to therapy. In the present study, we investigated an alternative immunohistochemistry (IHC)-based approach to achieve a molecular classification for GBM. For this purpose, a cohort of 100 surgical GBM samples was retrospectively evaluated by immunohistochemical analysis of EGFR, PDGFRA and p53. The quantitative analysis of these immunostainings allowed us to identify the following two GBM subtypes: the “Classical-like” (CL) subtype, characterized by EGFR-positive and p53- and PDGFRA-negative staining and the “Proneural-like” (PNL) subtype, characterized by p53- and/or PDGFRA-positive staining. This classification represents an independent prognostic factor in terms of overall survival compared to age, extent of resection and adjuvant treatment, with a significantly longer survival associated with the PNL subtype. Moreover, these two GBM subtypes exhibited different responses to chemotherapy. The addition of temozolomide to conventional radiotherapy significantly improved the survival of patients belonging to the CL subtype, but it did not affect the survival of patients belonging to the PNL subtype. We have thus shown that it is possible to differentiate between different clinically relevant subtypes of GBM by using IHC-based profiling, a method that is advantageous in its ease of daily implementation and in large-scale clinical application.

## Introduction

Glioblastoma (GBM), which represents the highest grade of glioma, is the most common malignant primary brain tumor in adults. Despite advances in the management of these tumors, GBMs are associated with poor prognosis and a median overall survival of only 14 months [1]. GBMs are considered by the World Health Organization classification as a single histological entity. However, GBMs are heterogeneous tumors that are characterized by considerable variability in biological behavior, which gives rise to significantly different prognoses and responses to treatment [1]. Abundant research on gliomas has identified molecular markers that are unique to specific histological types or to different grades of malignancy. Some of these markers have diagnostic value, whereas others are useful prognostic factors for patient survival or response to treatment [2], [3], [4]. However, the number of clinically relevant markers for GBM remains very limited. Frequent Isocitrate Dehydrogenase 1 (IDH1) mutations have been shown to be a prognostic marker that is associated with longer overall survival, but these mutations are almost exclusively restricted to secondary GBMs, which represent a minority of GBM cases [5], [6], [7]. The methylation of the O-6-methylguanine-DNA methyltransferase (MGMT) promoter until now has been the only predictive marker of the response of GBMs to treatment [8].

In an attempt to better understand GBM biology and to identify new clinically relevant markers, many groups have performed large-scale profiling studies based on gene or protein expression [9], [10], [11], [12], [13]. These studies have been used to identify subtypes of gliomas based on transcriptional or proteomic signatures. Interestingly, despite differences in the histological types of gliomas evaluated and in the data analysis process, two to four major subtypes consistently appear to emerge from these studies [14]. Although no clear consensus has been made in terms of these two to four subtypes, almost all of the studies identified a key distinction between subtypes with features that are described as proneural, mesenchymal and proliferative [14]. Interestingly, these different subtypes are associated with different prognoses or responses to therapy [9], [10], [12].

Recently, The Cancer Genome Atlas (TCGA) Research Network has generated a comprehensive catalog of genomic abnormalities in a large cohort of GBMs [11], [12], [15]. Verhaak et *al*. have used the TCGA data to correlate gene expression-based GBM subtypes with alterations in DNA sequences and copy numbers. They have thereby established a classification of GBM into Classical, Mesenchymal, Proneural and Neural subtypes and demonstrated that these subtypes are associated with specific genomic alterations. For instance, the Classical subtype is characterized by Epidermal Growth Factor Receptor (EGFR) amplification and the absence of p53 mutations. The Proneural subtype, on the other hand, is characterized by IDH1 and p53 mutations and Platelet Derived Growth Factor A (PDGFRA) amplification. Finally, the Mesenchymal subtype is characterized by deletions or mutation of the Neurofibromin 1 gene (NF1) [12]. Interestingly, using a proteomic approach, Brennan et *al*. have also shown that gliomas can be divided into three subtypes associated with EGFR activation, PDGFR activation and NF1 loss [11]. Such integrated work has thus revealed that GBMs can be classified into a few major subtypes on the basis of a small number of molecular aberrations [14].

In the present study, we investigated the possibility of classifying GBMs by evaluating the status of only three markers by using a method that is easily applicable in daily practice. For this purpose, we analyzed the expression of EGFR, PDGFRA and p53 using immunohistochemistry (IHC) on a retrospective cohort of 100 GBM surgical samples.

## Materials and Methods

### Ethics Statement

This work has been approved by the ethical committee of the Erasme University Hospital (Brussels, Belgium). According to the Belgian law of December 2008 « Loi relative à l'obtention et à l'utilisation de matériel corporel humain destiné à des applications médicales humaines ou à des fins de recherche scientifique », no written informed consent was required. The ethical committee has thus waived the need for written informed consent from the participant.

### Patients and tissue samples

Tissue samples were obtained retrospectively from archival formalin-fixed and paraffin-embedded samples from 100 GBMs collected between 2000 and 2005 in the department of Pathology of the Erasme Hospital, Brussels, Belgium. This time period was chosen in order to have a follow up of at least five years for all the patients. All of the tumors are from patients who were not previously treated for brain tumors (primitive glioblastomas) and whose histopathological diagnoses were reviewed by two pathologists (SR and IS) to ensure consistent diagnoses and tumor grading based on the 2007 WHO classification guidelines [1]. For each patient, two paraffin blocks containing representative tumor tissues were selected for analysis. The available clinical data for each patient were collected and included age at diagnosis, gender, tumor site, multifocality, the extent of resection, adjuvant treatment and follow-up (Table 1). The adjuvant treatments were classified into the four following groups: no treatment (including palliative management), radiotherapy alone, radiotherapy combined with temozolomide and others non-standard treatment. Patient outcomes were characterized in terms of progression-free survival and overall survival. The recurrences were defined as the cases, which by magnetic resonance imaging, presented evidence of progression that required a second resection or adjuvant treatments. Progression-free survival and overall survival were measured from the date of tumor resection until the date of recurrence or death (progression-free survival) or the date of death due to tumor progression (overall survival).

**Table 1:** Patients' demographics and features.

*Number of patients*	100
*Age (years)*	
Range	5.1–81.1
Median	64.8
Average	61.1
≤60	35
>60	65
*Gender*	
Female	60
Male	40
*Multifocality*	
Yes	19
No	81
*Extent of resection*	
complete	35
partial	65
*Adjuvant Therapy*	
No^a^	13
Radiotherapy	68
Radiotherapy + Temozolomide	17
Others^b^	2
*Overall survival (months)*	
Range	0.2–54.6
Median	9.6
*Progression-free survival (months)*	
Range	0.1–36.5
Median	3.9
Recurrence	99%
**Death**	98%

a Including no treatment and palliative management

b Including non-standard therapy such as chemotherapy alone or radiotherapy combined with a chemotherapy other than temozolomide.

### Immunohistochemistry

Five-μm-thick sections were subjected to standard IHC as previously described [16], [17]. The IHC expression was visualized by means of streptavidin-biotin-peroxidase complex kit reagents (BioGenex, San Ramon, CA, USA) using diaminobenzidine/H_2_O_2_ as the chromogenic substrate. Counterstaining with haematoxylin concluded the processing. The expression of EGFR, p53 and PDGFRA was detected by immunostaining using a mouse monoclonal anti-EGFR antibody (clone 31G7, dilution 1/500, Invitrogen, Carlsbad, USA), a mouse monoclonal anti-p53 antibody (clone DO-7, dilution 1/400, Dako, Glostrup, Denmark) and a rabbit polyclonal anti-PDGFRA antibody (dilution 1/50, Thermo Scientific, Waltham, USA). For each staining, an external positive control was included, as well as a negative control, which entailed replacing the primary antibody with non-immune serum (Dako, Glostrup, Denmark). In addition, anti-vimentin and anti-GFAP immunostainings were performed as controls to evaluate eventual fixation problems [18].

### Quantitative analysis of the immunostainings

All of the immunostained slides were immediately scanned using a NanoZoomer slide scanner (Hamamatsu, Hamamatsu, Japan). For each slide, immunostaining quality was controlled and the tumor bulk areas were selected by a pathologist (IS) using the NDP.view software (Hamamatsu, Hamamatsu, Japan). We limited our analysis to the tumor bulk area in order to avoid contamination by normal cells during quantification. Quantitative analysis of each immunostained area selected was performed using the Visiomorph software package (Visiopharm, Hoersholm, Denmark). For each GBM sample and for each staining we computed the labeling index (LI) and the Quick score (QS). The LI corresponds to the percentage of the immunostained tissue area and the QS to the global average pixel intensity, where the negative pixels were considered as a null intensity [18]. As detail in the results (for sake of clarity), in order to use the QS as a classification criteria, the quantitative values were dichotomized into “negative” and “positive” groups by comparing them to a semi-quantitative scoring.

### Statistical analysis

All of the statistical analyses were performed using Statistica (Statsoft, Tulsa, USA). Comparisons between two independent groups of numerical data were performed using the non-parametric Mann-Whitney test. The association between two binary variables was assessed using the Exact Fisher test. Univariate survival analyses were performed using the standard Kaplan–Meier analysis and the Wilcoxon-Gehan test, except in cases of continuous variables, for which univariate Cox regression was used. We completed these analyses by using multivariate Cox regression. When analyzing the set of clinical variables, we selected those for which the univariate results indicated a p-value <0.05. We then tested the potential contributions of the biological variables (IHC) to the final “clinical” model by adding those for which the univariate results indicated a p-value <0.1.

For each statistical analysis, the cases presenting missing value(s) in the concerned variable(s) were omitted.

The results of this study are reported according to the recommendations for tumor marker prognostic studies (REMARK) [19], [20].

## Results

### Patient's characteristics

A total of 100 patients were included in this study (Table 1). The median age at diagnosis was 64.8 years (range: 5.1–81.1) and the male to female ratio was 1.5∶1. The median overall survival was 9.6 months (range: 0.2–54.6) and the median progression-free survival was 3.9 months (range: 0.1–36.5). The extent of resection was complete for 35 patients and partial for 65 patients. Due to the long period of inclusion (2000–2005), the patients did not receive uniform treatment. Sixty-eight patients were treated with radiotherapy alone and 18 were treated with combined chemoradiation therapy (17 with temozolomide and 1 with BCNU). The other 14 patients received either chemotherapy alone (1 patient), palliative management (4 patients), or no treatment (9 patients). We first analyzed the impact of clinical variables on overall and progression-free survival. Univariate survival analyses were done on the 100 patients included, excepted for the treatment for which only the patients who received radiotherapy alone or radiotherapy combined with Temozolomide were analyzed (n = 85; Table 1). These analyses revealed that older age was associated with reduced overall survival (p = 0.035), a trend that was particularly significant in patients older than 60 (p = 0.003). Multifocality was associated with reduced overall survival (p = 0.03) and reduced progression-free survival (p = 0.001). As expected, complete resection and the addition of temozolomide to radiotherapy were also associated with better prognosis in terms of overall survival (p = 0.002 and p = 0.007, respectively) and of progression-free survival (p = 0.02 and p = 0.004, respectively). We then performed multivariate survival analysis by combining the clinical variables for which the univariate results indicated a p-value <0.05. Table 2 shows the final models (n = 85 because of the treatment variable) from which multifocality was excluded because this variable is correlated with the extent of resection and did not contribute significant information to the multivariate model (p>0.1). In terms of overall survival, the final model revealed that complete resection (p = 0.040) and the addition of temozolomide to the radiotherapy (p = 0.014) are independent prognostic factors that are associated with longer survival. The addition of temozolomide to the radiotherapy is also an independent prognostic factor (p = 0.002) associated with longer progression-free survival (Table 2).

**Table 2:** Multivariate Analysis of Survival (Cox Proportional Hazards Regression Model) involving clinical variables.

	*Model P-value*	*Prognostic factor*	*Hazard Ratio*	*95% CI*	*P- value*
*Overall survival*	0.0010	Age	1.02	1.00–1.03	0.087
		Complete resection	0.62	0.39–0.98	0.040
		Radiotherapy + Temozolomide	0.48	0.27–0.86	0.014
					
*Progression-free survival*	0.0017	Age	1.00	0.98–1.02	0.747
		Complete resection	0.68	0.42–1.08	0.100
		Radiotherapy + Temozolomide	0.36	0.19–0.69	0.002

The Model P-value indicates the overall level of significance of the multivariate model. Aside from “Age”, which is a continuous variable, the other variables are binary. These variables distinguish between complete and partial resection and radiotherapy + temozolomide compared with radiotherapy alone. The individual P-values represent the levels of significance of the independent contributions of each factor.

CI: confidence interval

### PDGFRA, p53 and EGFR expression

We first analyzed the individual expression of EGFR, p53 and PDGFRA in our series of 100 GBM patient samples. For each marker, quantitative analyses were performed to evaluate the labeling index (LI), which corresponds to the percentage of the immunostained tissue area and the Quick score (QS), which corresponds to the global average pixel intensity. Quantitative data were obtained for 97 patients for EGFR and PDGFRA and for 95 patients for p53. When considered individually, no association was detected between the expression (LI and QS) of p53 or PDGFRA and either overall survival or progression-free survival (data not shown). Patients overexpressing EGFR exhibited a trend toward longer overall survival (p = 0.09 for the QS and p = 0.08 for the LI) and toward longer progression-free survival (p = 0.051 for both the QS and the LI). However, Table 3 shows that the QS of EGFR was not an independent prognostic factor in multivariate models involving the clinical variables. Similar results were obtained for the LI of EGFR (data not shown).

**Table 3:** Multivariate Analysis of Survival (Cox Proportional Hazards Regression Model) involving clinical variables and the QS of EGFR.

	*Model P-value*	*Prognostic factor*	*Hazard Ratio*	*95% CI*	*P- value*
*Overall survival*	0.00175	Age	1.02	1.00–1.03	0.086
		Complete resection	0.63	0.40–0.99	0.049
		Radiotherapy + Temozolomide	0.49	0.27–0.89	0.019
		EGFR QS	1.00	1.00–1.01	0.271
					
*Progression-free survival*	0.00214	Age	1.00	0.98–1.02	0.85
		Complete resection	0.63	0.39–1.03	0.065
		Radiotherapy + Temozolomide	0.38	0.20–0.72	0.003
		EGFR QS	1.01	1.00–1.01	0.174

The Model P-value indicates the overall level of significance of the multivariate model. Aside from “Age” and “EGFR QS”, which are continuous variables, the other variables are binary. These variables distinguish between complete and partial resection and radiotherapy + temozolomide compared with radiotherapy alone. The individual P-values represent the levels of significance of the independent contributions of each variable.

CI: confidence interval

**Figure 1:**
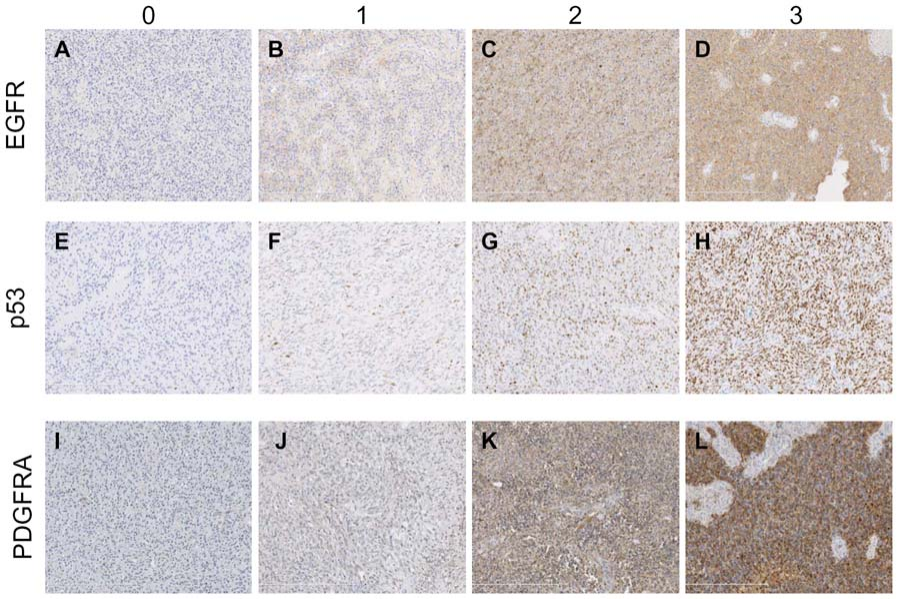
Immunohistochemical analysis of EGFR, p53 and PDGFRA expression in GBM patient samples. Distinct levels of protein expression detected by IHC for EGFR (A–D), p53 (E–H) and PDGFRA (I–L) in GBM patient samples. The panels illustrate cases graded according to score 0 (A, E and I), score 1 (B, F, J), score 2 (C, G and K) and score 3 (D, H and L) in the 4-tier semi-quantitative analyses.

To use these markers as classification criteria, the magnitude of expression detected by each immunostaining analysis was dichotomized into “negative” and “positive” groups. For this purpose, we have compared the QS values to a semi-quantitative 4-score (0, 1, 2 and 3) system (illustrated in Figure 1). The QS threshold was set to 20, which is the value that most effectively differentiates the 0 and 1 scores (labeled “negative”) compared to the 2 and 3 scores (labeled “positive) for the 3 markers (data not shown). Fifty-three percent of the GBM samples were considered positive for PDGFRA; 73% were considered positive for EGFR; and 21% were considered positive for p53. For each of the three markers, overall survival and progression-free survival were not significantly different between the positive and negative groups (data not shown).

### GBM subtypes

**Figure 2:**
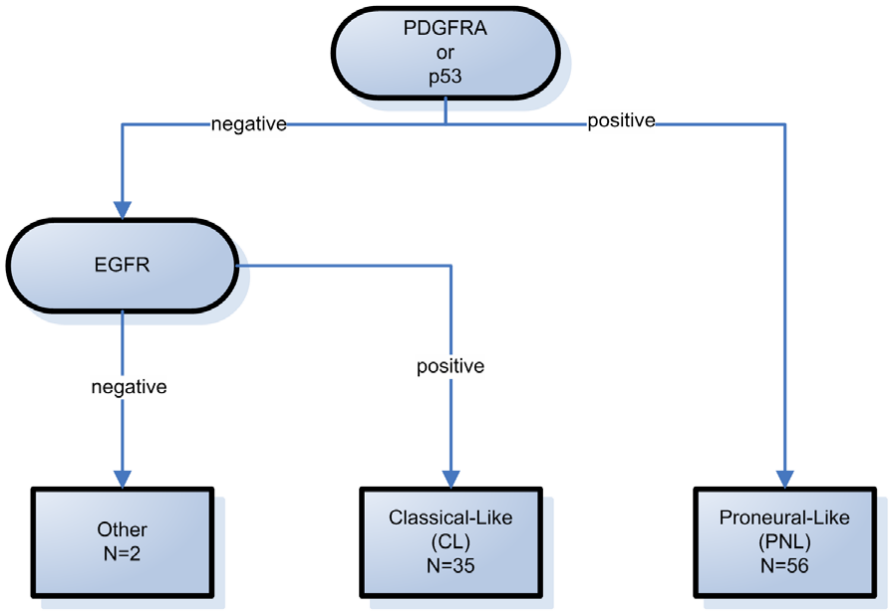
Algorithm used for the Classification of GBM cases into the different subtypes. p53- and/or PDGFRA-positive cases (n = 56) were labeled as Proneural-Like (PNL); p53- and PDGFRA-negative and EGFR-positive cases (n = 35) were labeled Classical-Like (CL). Cases negative for the three markers were labeled as “Other” (n = 2).

Inspired by the recent molecular classification described by Verhaak et *al*. (cf. Introduction), we investigated whether the different GBM subtypes could be identified on the basis of the three markers that we evaluated (after their binarization as described above). Figure 2 illustrates the algorithm that was used to classify the GBMs into two different subtypes. The first subtype, “Classical-Like” (CL), is characterized by EGFR-positive staining combined with both p53- and PDGFRA-negative staining. The second subtype, “Proneural-Like” (PNL), is characterized by p53- and/or PDGFRA-positive staining. The GBMs that did not fit any of these criteria were classified as “Other” (Figure 2). The clinical data for each subtype are summarized in Table 4. Among the 100 GBMs analyzed, quantitative analyses of the three markers were available for 93 GBMs; 35 (37.6%) were classified in the CL subtype and 56 (60.2%) were classified in the PNL subtype. Two GBMs (2.2%) were classified as “Other”; both were negative for the three markers. The age of the patients at diagnosis was not significantly different between the CL and PNL subtypes (cf. Table 4; p = 0.18). No significant association was detected between the subtypes (PNL vs. CL) and gender (p = 0.38), multifocality (p = 0.42), the extent of resection (p = 0.51) and the type of treatment (radiotherapy alone vs. radiotherapy + temozolomide; p = 0.08; n = 85).

**Table 4:** Patients' demographics and features in the different subtypes.

	*Classical-Like*	*Proneural-Like*	*Other*
*N*	35	56	2
*Age (years)*			
Range	5.1–81.1	21.2–77.5	52.9–69.7
Median	65.9	63.5	61.3
Average	62.8	59.9	61.3
≤60	10	21	1
>60	25	35	1
*Gender*			
Female	11	23	1
Male	24	33	1
*Multifocality*			
Yes	8	9	1
No	27	47	1
*Extent of resection*			
complete	11	22	1
partial	24	34	1
*Adjuvant Therapy*			
No^a^	6	4	1
Radiotherapy	19	44	0
Radiotherapy + Temozolomide	9	7	1
Others^b^	1	1	0
*Overall survival (months)*			
Range	0.6–54.6	0.2–40.0	2.7–35.9
Median	5.0	10.5	19.3
*Progression-free survival (months)*			
Range	0.5–24.3	0.1–36.5	2.7–28.2
Median	3.6	4.3	15.4
Recurrence	100%	98%	100%
**Death**	100%	96%	100%

The table displays the numbers (or percentages) of cases within the different GBM subtypes, except where other features are indicated (such as range or median).

a Including no treatment and palliative management

b Including non-standard therapy such as chemotherapy alone or radiotherapy combined with a chemotherapy other than temozolomide.

### Prognostic significance of the GBMs classification

**Figure 3:**
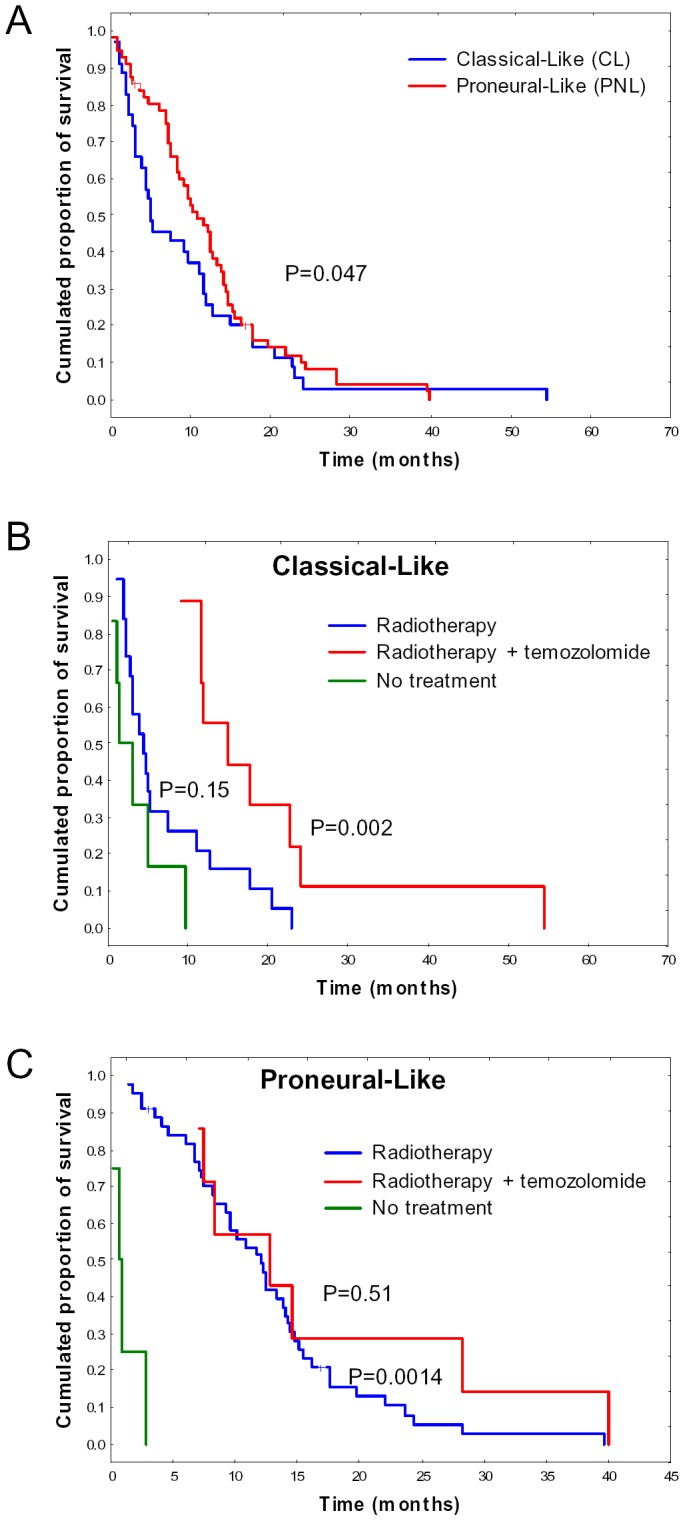
Kaplan-Meier Survival curves of GBM patients dichotomized according to PNL vs. CL classification. (A) The overall survival was significantly higher for GBM patients of PNL subtype compared to patients of CL subtype (p = 0.047). (B) For GBM patients of the CL subtype, the addition of temozolomide to radiotherapy significantly improved the patient's overall survival (p = 0.002), but radiotherapy alone exhibited no significant effect when compared with palliative management or no treatment (p = 0.15). (C) For GBM patients of the PNL subtype, radiotherapy alone significantly improved the overall patient's survival when compared to palliative management or no treatment (p  =  0.0014), but the addition of temozolomide conferred no significant effect when compared to radiotherapy alone (p = 0.51).

Figure 3A shows that the overall survival was significantly higher for the patients belonging to the PNL subtype than those belonging to the CL subtype (p = 0.047). We then performed a multivariate survival analysis by combining the GBMs classification with the clinical variables used in Table 2 (age, extent of resection and adjuvant treatment). This analysis was done on the patients belonging to the CL or PNL subtypes and who received radiotherapy alone or radiotherapy combined with Temozolomide (n = 79). Table 5 shows that older age (p = 0.022), the addition of temozolomide to radiotherapy (p = 0.002) and classification in the PNL subtype (p = 0.008) are all independent prognostic factors associated with longer overall survival. There was a mortality risk reduction of 52% associated with the PNL subtype compared to the CL subtype (hazard ratio of 0.48).

**Table 5:** Multivariate Analysis of Survival (Cox Proportional Hazards Regression Model) involving clinical variables and GBMs classification.

	*Model P-value*	*prognostic factor*	*Hazard Ratio*	*95% CI*	*P- value*
*Overall survival*	0.00052	Age	1.02	1.00–1.04	0.022
		Complete resection	0.63	0.40–1.02	0.060
		Radiotherapy + temozolomide	0.37	0.19–0.70	0.002
		PNL subtype	0.48	0.29–0.83	0.008

The Model P-value indicates the overall level of significance of the multivariate model. Aside from “Age” which is a continuous variable, the other variables are binary. These variables distinguish between complete and partial resection, radiotherapy + temozolomide compared with radiotherapy alone, as well as the classification into the PNL subtype or into the CL subtype. The individual P-values represent the levels of significance of the independent contributions of each variable.

CI: confidence interval

We then analyzed whether the response to treatment was the same in the two GBM subtypes. As shown in Figure 3B, the addition of temozolomide to radiotherapy significantly improved the overall survival of the patients belonging to the CL subtype (p = 0.002). However, radiotherapy alone did not improve the survival of the patients with this GBM subtype when compared with palliative management or no treatment (p = 0.15). In contrast, whereas radiotherapy alone significantly improved the overall survival of the patients belonging to the PNL subtype compared to palliative management or no treatment (p = 0.0014), the addition of temozolomide exhibited no significant effect for the patients of this GBM subtype (p = 0.51; Figure 3C).

## Discussion

Although GBMs are morphologically defined as a unique entity, they are extremely heterogeneous tumors associated with great variability in terms of response to treatment and clinical outcome [1]. Thus, there is a need to improve the morphologically based classification of these tumors with the integration of molecular data. In the last decade, several studies have been performed to identify subtypes of gliomas based on transcriptional signatures [14]. It is now clear that these signatures can convey information about prognosis. However, because these molecular classifications are based on high-throughput genomics and proteomics technologies, none of these signatures are amenable for daily practice. These technologies are usually complex, expensive, time consuming and are not widely available. Furthermore, these techniques generate a large amount of data that require extensive validation and are difficult to apply to individual samples [21]. Finally these transcriptomic analyses usually require the use of frozen tissue, which are not always available.

In the present study, we have shown that GBMs can be classified into two clinically relevant subtypes by using IHC-based evaluation of only three markers—a simple method applicable in daily practice. IHC-based techniques offer many advantages. First, this technique is optimized for formalin-fixed and paraffin-embedded tissue, which is the standard approach for processing tumor tissue in pathology laboratories worldwide. Other examples of molecular signatures identified by gene expression profiling technologies have already been validated at the protein level by IHC and implemented in clinical laboratories [22]. Moreover, IHC-based evaluation is fast and requires only a small amount of tissue, allowing the report of the results within the rapid turn-around time expected by clinicians and patients, even for small surgically resected specimens. Because IHC analyses are performed on tissue sections in which architectural features are conserved, IHC-based evaluation also allows for the advantageous examination of the spatial heterogeneity of protein expression. This cellular heterogeneity is lost using genomic and proteomic methods, which requires the homogenization of the tissue samples. Finally, quantification of the immunostaining can be standardized with the use of calibrated image acquisition using quantification software packages [18], [23], [24]. However, standardized protocols of tissue fixation and adapted quality control are crucial for avoiding technique-derived variation in IHC stainings and in their subsequent quantification [18], [25], [26].

Based on the study of Verhaak et *al*., we chose to analyse the expression of p53, EGFR and PDGFRA in order to identify two GBM subtypes that we labelled, “Proneural-like” and “Classical-like” (see Figure 2). Our approach was motivated by the observation that p53 is often mutated in patients of the Proneural subtype but not in the Classical subtype. EGFR amplification characterizes the Classical subtype, whereas PDGFRA amplification is more specific to the Proneural subtype [12]. The correlation between p53 mutations and p53 IHC expression remains debatable. However, the overexpression of p53 in GBM is often associated with the presence of p53 mutations [27], [28], [29]. In contrast, the correlation between EGFR amplification and overexpression detected by IHC is well documented [30], [31], [32], [33]. Finally, whereas no correlation was detected between PDGFR amplification and IHC expression [34], the Proneural subtype defined by Verhaak et *al*. was characterized by the presence of PDGFRA amplification in conjunction with high levels of gene expression [12]. On this basis, we defined the “Proneural-like” (PNL) subtype, which is characterized by p53- and/or PDGFRA-positive staining and the “Classical-like” (CL) subtype, which is characterized by EGFR-positive staining and the absence of both p53 and PDGFRA staining. We show that this classification constitutes an independent prognostic factor in terms of overall survival compared to age, extent of resection and adjuvant treatment. Moreover, these two subtypes of GBM exhibit different responses to chemotherapy. It is interesting to note that we found no association between patient outcomes and the IHC expression of each marker characterized individually. These latter results are consistent with previously published data [30], [34], [35], [36] and suggest that biomarkers should be combinatorially analyzed as a signature rather than individually.

In the Verhaak et *al*'s classification, the Proneural subtype is characterized by PDGFRA amplification, p53 mutation and IDH1 mutations. We were able to analyse IDH1 mutations in 66 GBMs (data not shown) from which only 2 (3%) exhibited IDH1 mutations. This small number can be explained by the fact that we selected patients with untreated GBMs before resection, thereby reducing the number of secondary GBMs, which typically carry IDH1 mutations [5]. Consistent with published data showing the existence of a correlation between p53 and IDH1 mutations [5], [37], both IDH1 mutated cases were also positive for p53 and were thus labelled as “Proneural-like” in our classification.

In addition to the Proneural and the Classical subtypes, Verhaak et *al*. described two other subtypes, Neural and Mesenchymal. Whereas the Neural subtype is not well characterized, deletion and/or mutation of NF1 and low levels of NF1 gene expression are typical of the Mesenchymal subtype [5]. This subtype has already been previously described in other studies and is associated with poor prognosis [9], [10], [14]. Thus, it would be interesting to include in our classification another marker, such as NF1, MET or YKL-40, which are characteristic of this class of GBM [9], [10], in order to derive a third subtype, thereby improving the clinical significance of our classification.

In conclusion, we have demonstrated that the IHC-based analysis of three markers, p53, EGFR and PDGFRA, allowed us to identify two GBM subtypes with prognostic significance in terms of overall survival and response to treatment. This study thus shows that quantitative immunohistochemistry involving only three biomarkers is sufficient to identify clinically relevant subtypes of GBM. This advantageous approach can be easily applicable in daily practice and allows for large scale-clinical application.

## References

[1] Louis DN, Ohgaki H, Wiestler OD, Cavenee WK (2007) WHO classification of tumours of the central nervous system; Bosman FT, Jaffe ES, Lakhani SR, Ohgaki H, editors. Lyon: International Agency for Research on Cancer (IARC).

[2] Nikiforova MN, Hamilton RL (2011) Molecular diagnostics of gliomas. Arch Pathol Lab Med 135: 558–568. 21526954 10.5858/2010-0649-RAIR.1

[3] Ducray F, El Hallani S, Idbaih A (2009) Diagnostic and prognostic markers in gliomas. Curr Opin Oncol 21: 537–542. 19667985 10.1097/CCO.0b013e32833065a7

[4] Riemenschneider MJ, Jeuken JW, Wesseling P, Reifenberger G (2010) Molecular diagnostics of gliomas: state of the art. Acta Neuropathol 120: 567–584. 20714900 10.1007/s00401-010-0736-4 PMC2955236

[5] Nobusawa S, Watanabe T, Kleihues P, Ohgaki H (2009) IDH1 mutations as molecular signature and predictive factor of secondary glioblastomas. Clin Cancer Res 15: 6002–6007. 19755387 10.1158/1078-0432.CCR-09-0715

[6] Sanson M, Marie Y, Paris S, Idbaih A, Laffaire J, et al (2009) Isocitrate dehydrogenase 1 codon 132 mutation is an important prognostic biomarker in gliomas. J Clin Oncol 27: 4150–4154. 19636000 10.1200/JCO.2009.21.9832

[7] Yan H, Parsons DW, Jin G, McLendon R, Rasheed BA, et al (2009) IDH1 and IDH2 mutations in gliomas. N Engl J Med 360: 765–773. 19228619 10.1056/NEJMoa0808710 PMC2820383

[8] Hegi ME, Diserens AC, Gorlia T, Hamou MF, de Tribolet N, et al (2005) MGMT gene silencing and benefit from temozolomide in glioblastoma. N Engl J Med 352: 997–1003. 15758010 10.1056/NEJMoa043331

[9] Freije WA, Castro-Vargas FE, Fang Z, Horvath S, Cloughesy T, et al (2004) Gene expression profiling of gliomas strongly predicts survival. Cancer Res 64: 6503–6510. 15374961 10.1158/0008-5472.CAN-04-0452

[10] Phillips HS, Kharbanda S, Chen R, Forrest WF, Soriano RH, et al (2006) Molecular subclasses of high-grade glioma predict prognosis, delineate a pattern of disease progression, and resemble stages in neurogenesis. Cancer Cell 9: 157–173. 16530701 10.1016/j.ccr.2006.02.019

[11] Brennan C, Momota H, Hambardzumyan D, Ozawa T, Tandon A, et al (2009) Glioblastoma subclasses can be defined by activity among signal transduction pathways and associated genomic alterations. PLoS One 4: e7752. 19915670 10.1371/journal.pone.0007752 PMC2771920

[12] Verhaak RG, Hoadley KA, Purdom E, Wang V, Qi Y, et al (2010) Integrated genomic analysis identifies clinically relevant subtypes of glioblastoma characterized by abnormalities in PDGFRA, IDH1, EGFR, and NF1. Cancer Cell 17: 98–110. 20129251 10.1016/j.ccr.2009.12.020 PMC2818769

[13] Nutt CL, Mani DR, Betensky RA, Tamayo P, Cairncross JG, et al (2003) Gene expression-based classification of malignant gliomas correlates better with survival than histological classification. Cancer Res 63: 1602–1607. 12670911

[14] Huse JT, Phillips HS, Brennan CW (2011) Molecular subclassification of diffuse gliomas: Seeing order in the chaos. Glia. 10.1002/glia.21165 21446051

[15] TCGA TCGARN (2008) Comprehensive genomic characterization defines human glioblastoma genes and core pathways. Nature 455: 1061–1068. 18772890 10.1038/nature07385 PMC2671642

[16] D'Haene N, Maris C, Sandras F, Dehou MF, Remmelink M, et al (2005) The differential expression of Galectin-1 and Galectin-3 in normal lymphoid tissue and non-Hodgkin's and Hodgkin's lymphomas. Int J Immunopathol Pharmacol 18: 431–443. 16164826 10.1177/039463200501800304

[17] Rorive S, Lopez XM, Maris C, Trepant AL, Sauvage S, et al (2010) TIMP-4 and CD63: new prognostic biomarkers in human astrocytomas. Mod Pathol 23: 1418–1428. 20693981 10.1038/modpathol.2010.136

[18] Decaestecker C, Lopez XM, D'Haene N, Roland I, Guendouz S, et al (2009) Requirements for the valid quantification of immunostains on tissue microarray materials using image analysis. Proteomics 9: 4478–4494. 19670370 10.1002/pmic.200800936

[19] McShane LM, Altman DG, Sauerbrei W, Taube SE, Gion M, et al (2005) Reporting recommendations for tumor marker prognostic studies (REMARK). J Natl Cancer Inst 97: 1180–1184. 16106022 10.1093/jnci/dji237

[20] Altman DG, McShane LM, Sauerbrei W, Taube SE (2012) Reporting Recommendations for Tumor Marker Prognostic Studies (REMARK): explanation and elaboration. PLoS Med 9: e1001216. 22675273 10.1371/journal.pmed.1001216 PMC3362085

[21] McShane LM, Altman DG, Sauerbrei W (2005) Identification of clinically useful cancer prognostic factors: what are we missing? J Natl Cancer Inst 97: 1023–1025. 16030294 10.1093/jnci/dji193

[22] Muris JJ, Meijer CJ, Vos W, van Krieken JH, Jiwa NM, et al (2006) Immunohistochemical profiling based on Bcl-2, CD10 and MUM1 expression improves risk stratification in patients with primary nodal diffuse large B cell lymphoma. J Pathol 208: 714–723. 16400625 10.1002/path.1924

[23] Metzger GJ, Dankbar SC, Henriksen J, Rizzardi AE, Rosener NK, et al (2012) Development of multigene expression signature maps at the protein level from digitized immunohistochemistry slides. PLoS One 7: e33520. 22438942 10.1371/journal.pone.0033520 PMC3305321

[24] Rizzardi AE, Johnson AT, Isaksson Vogel R, Pambuccian SE, Henriksen J, et al (2012) Quantitative comparison of immunohistochemical staining measured by digital image analysis versus pathologist visual scoring. Diagn Pathol 7: 42. 22515559 10.1186/1746-1596-7-42 PMC3379953

[25] Kajdacsy-Balla A, Geynisman JM, Macias V, Setty S, Nanaji NM, et al (2007) Practical aspects of planning, building, and interpreting tissue microarrays: the Cooperative Prostate Cancer Tissue Resource experience. J Mol Histol 38: 113–121. 17318343 10.1007/s10735-006-9054-5

[26] Eguiluz C, Viguera E, Millan L, Perez J (2006) Multitissue array review: a chronological description of tissue array techniques, applications and procedures. Pathol Res Pract 202: 561–568. 16782284 10.1016/j.prp.2006.04.003

[27] Newcomb EW, Madonia WJ, Pisharody S, Lang FF, Koslow M, et al (1993) A correlative study of p53 protein alteration and p53 gene mutation in glioblastoma multiforme. Brain Pathol 3: 229–235. 8293182 10.1111/j.1750-3639.1993.tb00749.x

[28] Yoon KS, Lee MC, Kang SS, Kim JH, Jung S, et al (2001) p53 mutation and epidermal growth factor receptor overexpression in glioblastoma. J Korean Med Sci 16: 481–488. 11511795 10.3346/jkms.2001.16.4.481 PMC3054785

[29] Pollack IF, Hamilton RL, Finkelstein SD, Campbell JW, Martinez AJ, et al (1997) The relationship between TP53 mutations and overexpression of p53 and prognosis in malignant gliomas of childhood. Cancer Res 57: 304–309. 9000573

[30] Lopez-Gines C, Gil-Benso R, Ferrer-Luna R, Benito R, Serna E, et al (2010) New pattern of EGFR amplification in glioblastoma and the relationship of gene copy number with gene expression profile. Mod Pathol 23: 856–865. 20305620 10.1038/modpathol.2010.62

[31] Marquez A, Wu R, Zhao J, Tao J, Shi Z (2004) Evaluation of epidermal growth factor receptor (EGFR) by chromogenic in situ hybridization (CISH) and immunohistochemistry (IHC) in archival gliomas using bright-field microscopy. Diagn Mol Pathol 13: 1–8. 15163002 10.1097/00019606-200403000-00001

[32] Hatanpaa KJ, Burma S, Zhao D, Habib AA (2010) Epidermal growth factor receptor in glioma: signal transduction, neuropathology, imaging, and radioresistance. Neoplasia 12: 675–684. 20824044 10.1593/neo.10688 PMC2933688

[33] Coulibaly B, Nanni I, Quilichini B, Gaudart J, Metellus P, et al (2010) Epidermal growth factor receptor in glioblastomas: correlation between gene copy number and protein expression. Hum Pathol 41: 815–823. 20303140 10.1016/j.humpath.2009.09.020

[34] Martinho O, Longatto-Filho A, Lambros MB, Martins A, Pinheiro C, et al (2009) Expression, mutation and copy number analysis of platelet-derived growth factor receptor A (PDGFRA) and its ligand PDGFA in gliomas. Br J Cancer 101: 973–982. 19707201 10.1038/sj.bjc.6605225 PMC2743351

[35] Ohgaki H, Dessen P, Jourde B, Horstmann S, Nishikawa T, et al (2004) Genetic pathways to glioblastoma: a population-based study. Cancer Res 64: 6892–6899. 15466178 10.1158/0008-5472.CAN-04-1337

[36] Rasheed A, Herndon JE, Stenzel TT, Raetz JG, Kendelhardt J, et al (2002) Molecular markers of prognosis in astrocytic tumors. Cancer 94: 2688–2697. 12173338 10.1002/cncr.10544

[37] Ichimura K, Pearson DM, Kocialkowski S, Backlund LM, Chan R, et al (2009) IDH1 mutations are present in the majority of common adult gliomas but rare in primary glioblastomas. Neuro Oncol 11: 341–347. 19435942 10.1215/15228517-2009-025 PMC2743214

